# Editorial: Bridging Reading Aloud and Speech Production

**DOI:** 10.3389/fpsyg.2016.00661

**Published:** 2016-05-06

**Authors:** Simone Sulpizio, Sachiko Kinoshita

**Affiliations:** ^1^Department of Psychology and Cognitive Science, University of TrentoTrento, Italy; ^2^Fondazione Marica De Vincenzi ONLUSRovereto, Italy; ^3^Department of Psychology, Macquarie UniversitySydney, NSW, Australia

**Keywords:** reading aloud, speech production, phonological encoding, lexical access, planning, bilingualism, ERPs, eye-tracking

The study of how people can speak or read started from the beginning of the modern era of psycholinguistics and neurolinguistics (e.g., Lichteim, 1885; Huey, 1898) and continues to this day. However, the two lines of research—speech production and reading aloud—have followed two separate and parallel paths: While they both concern language production, they seldom meet. Both have produced detailed descriptions of cognitive mechanisms underlying the processes that goes from the message planning to its articulatory realization, but they generally have little contact with each other.

Given their long and fruitful history, this parallel and separate courses of the two research traditions is quite surprising. Everyday experience suggests that speech production and reading aloud do have something in common: In both cases the endpoint is to utter the same linguistic units (saying the word table and reading aloud TABLE will produce the same acoustic material). This suggests that the processes of speech production and reading aloud may not be totally independent: There must be some shared processes at least in terms of generation of phonology and preparation of a motor speech response. The aim of the present research topic is to point the magnifying glass on this issue and to address questions such as the following: To what extent are speech production and word reading/reading aloud similar? Are there some shared components and/or mechanisms between the two process? Is the time course of the (supposed) shared mechanisms activation similar in the two processes? How does the different input (conceptual vs. orthographic) interact with the types of information that reading and speaking share?

Our call has resulted in 12 excellent articles (9 original research, 1 mini review, 1 opinion, 1 perspective article) that provide a first answer to the above questions and provide the impetus for future research. Three articles address the issue of similarity and differences between the processing stages and components of speech production and reading. Valente et al. inspected the spatio-temporal segmentation of ERPs in response to picture naming and word reading and shown that the two tasks are highly similar from 250 ms onward, which is an index of a shared phonological processing stage. Topographic similarities emerged also between 75 and 150 ms, suggesting similar visual processes, although of variable intensity, between the two tasks. Converging evidence for a shared phonological processing comes from Givon and Friedmann who studied patients with phonological output lexicon anomia: They demonstrate that lexical retrieval and reading are tightly linked processes, and suggest a principled relation between dyslexia and anomia. In contrast, Navarrete et al. highlight a difference between reading aloud and speech production: Testing semantic context effects, the authors shown that these effects can be transferred from pictures to words, but not *vice versa*, since the format of the stimuli affects lexical retrieval. As well as contributing to theoretical advancement, the Navarrete et al.'s findings have important methodological implication for the study of lexical access in speech production, when selecting stimulus materials.

Four original research articles targeted the stage of message planning. Speech production and reading aloud are both incremental processes, in which people tend to plan and articulate chunks that are smaller than the whole message they want to utter. Ganushchak and Chen ran two eye-tracking experiments to investigate how the utterance planning is affected by linguistic information (known vs. new information) in reading (sentence reading) vs. speaking (picture description) tasks and showed that planning is more incremental during reading than speaking and that this difference may be ascribed to conceptual preparation. Focusing on words, Mousikou and Rastle investigated participants' response in reading aloud and picture naming to high- and low-frequency stimuli by examining response latencies, and initial-phoneme and whole-word durations. Response latencies were shorter in reading than in picture naming, but initial-phoneme and whole-word durations were longer in the former than in the latter: These findings indicate that reading aloud, but not picture naming, is initiated on the basis of partial information from the printed word, and, that the effect of higher-level cognitive processes influence, to some extent, lower-level articulatory processes. Similarly, Sulpizio and Job offer evidence for a rapid initiation of articulation in reading aloud: By manipulating segmental and suprasegmental information in a series of masked-priming experiments, the authors shown that articulation planning is addressed through a process that starts as soon as the relevant information about the to-be-planned unit (i.e., stress position and phonemes of the stressed syllable) is active. An even extreme position is offered by the Kawamoto et al.'s mini-review, in which the authors advance the intriguing proposal that the minimal planning unit in reading aloud *and* speech production is the single initial segment.

Two original research articles looked at reading and speaking in bilinguals. Reynolds et al. investigated asymmetric switch costs in unbalanced bilinguals performing production and comprehension tasks: They shown that switch costs are affected by the task participants perform, highlighting that there exist relevant task-related differences in how different languages are controlled. Nakayama et al. investigated the phonological encoding of low- and high-proficient Japanese-English bilinguals, two languages that differ in the functional phonological unit used in speech production: Their results show that, when processing L2 English, low-proficient bilinguals keep using the phonological unit of their L1, whereas high-proficient bilinguals can use that of the L2. Moreover, their results suggested that it is the length of exposure to L2, rather than Age of Acquisition or proficiency, that led to the adoption of more native-like unit of phonological encoding.

The article by Laubrock and Kliegl is a substantial investigation of the eye-voice span in reading, and its relation with the eye-movement behavior and the response dynamics: As well as offering a promising direction for the understanding of the eye-voice coordination in reading, the study shows that the eye-voice span is directly related to the process of working-memory buffer updating. Finally, the opinion and the perspective article close the topic advancing intriguing theoretical proposal. Schmalz et al. propose a new solution for the issue of lexical selection that can account for performance in tasks as Stroop, picture-word and word-word interference: In their proposal, the conflict is resolved by linking the stimulus perceptual features with the linguistic information, which allows the system to identify which is the target and which is the distractor. Finally, Saletta proposes that speaking and reading are tightly linked since they share mechanisms of processing and learning; she argues that orthographic input exerts positive effects on speech learning and evocatively links these effects to individual's speech movements and motor control.

Overall, we believe that this Research Topic provides a good start to our initial questions. By means of the use of different techniques and the involvement of different populations, the empirical articles highlights similarities and differences between reading aloud and speech production; at the same time, theoretical articles offer new perspectives that guide the direction for future research. Altogether, the articles offer a solid scaffold for the challenge of bridging reading aloud and speech production. We hope that future research will build on the scaffold and pursue this line by filling the remaining gaps that mutually benefit the research on speech production and reading.

## Author contributions

SS and SK have made substantial, direct and intellectual contribution to the work, and approved it for publication.

### Conflict of interest statement

The authors declare that the research was conducted in the absence of any commercial or financial relationships that could be construed as a potential conflict of interest.
